# Anthropogenic Sewage Water Circuit as Vector for SARS-CoV-2 Viral ARN Transport and Public Health Assessment, Monitoring and Forecasting—Sibiu Metropolitan Area (Transylvania/Romania) Study Case

**DOI:** 10.3390/ijerph191811725

**Published:** 2022-09-17

**Authors:** Ioana Boeraș, Angela Curtean-Bănăduc, Doru Bănăduc, Gabriela Cioca

**Affiliations:** 1 Applied Ecology Research Center, Faculty of Sciences, Lucian Blaga University of Sibiu, 550012 Sibiu, Romania; 2 Preclinical Department, Faculty of Medicine, Lucian Blaga University of Sibiu, 550169 Sibiu, Romania

**Keywords:** sewage water, SARS-CoV-2, monitoring, risk assessment, management

## Abstract

Water is a risk factor for epidemics of waterborne diseases with effects on human health. In 2019, new viral pneumonia cases occurred in China and spread worldwide. The aim of this study was to assess the feasibility and accuracy of a wastewater-based epidemiological (WBE) monitoring tool in a SARS-CoV-2 hot spot (Sibiu City metropolitan area), namely to highlight the correlation between the number of infections on the days of sampling and the amount of viral RNA detected in wastewater. Wastewater samples were collected once a week, and viral RNA was extracted and quantified. In parallel, the daily number of SARS-CoV-2 infections was obtained from the local council. The correlation between the number of infections and viruses detected in sewage was measured by Pearson correlation coefficients. The results show the amount of viral RNA in the wastewater is directly correlated with the number of infections reported in the week up to the sampling day and also the number of infections reported for the sampling day. Moreover, correlation coefficients show the amount of viral RNA in wastewater increases in advance of the increase in reported infection cases. Therefore, WBE can be used as a tool for monitoring virus spread trends in human communities and can help anticipate the trend of this type of viral infection.

## 1. Introduction

In December 2019, a wave of unidentified viral pneumonia cases occurred in Wuhan, China, and quickly spread worldwide [1,2]. The virus causing this infection belonged to the family Coronaviridae and was named Severe Acute Respiratory Syndrome Coronavirus (SARS-CoV-2) [3]. The rapid spread of the SARS-CoV-2 virus led to the Coronavirus Disease 2019 (COVID-19) pandemic. The World Health Organization declared the outbreak a Public Health Emergency of International Concern on 30 January 2020 and a pandemic on 11 March 2020. As of June 2022, there were over 536 million infections and 6.31 million deaths worldwide, while in Romania, there have been 2.91 million infections and 65,714 deaths since the pandemic’s beginning. After over two years, this virus is still lurking in and around human communities around the globe, despite the enormous international efforts to control it.

SARS-CoV-2 viral infection is mainly spread through aerosols and infects the lungs causing respiratory symptoms. However, in a large number of infections, the virus has also been found to cause gastrointestinal tract symptoms [4,5] and to be shed in the faeces [6,7,8]. According to a retrospective cohort study, viral RNA was detected in the stool of 59% of patients with a median duration of viral RNA in the stool of 22 days [9]. The viral RNA load in stool samples reflected the course in sputum in 86% of cases in a German study [10]. In some patients, faecal samples remained positive for the virus even after the respiratory and/or sputum samples exhibited no detectable virus [10]. In some cases, faeces’ viral load was higher than that in pharyngeal swabs. The presence and persistence of such large amounts of viral RNA in faeces are unlikely to be explained by only swallowing virus particles replicated in the throat but instead suggest the potential for enteric infection of SARS-CoV-2 [11,12,13]. The presence of the virus in the faeces leads to the presence of the virus in the wastewater. Although less studied, the faecal–oral route of transmission has also been investigated [14,15,16].

Efforts to stop the pandemic have focused on stopping the spread of the virus, which requires early identification of new infections. Due to the symptoms being similar to other respiratory infections and the delayed onset of symptoms, detection of new infections and the needed management measures can be delayed. Therefore, alternative methods to detect new infection outbreaks early can be of help, as well as new potential ways/mediums of spreading. One such method is wastewater-based epidemiology (WBE), which has been successfully used in the past to identify and stop other viral outbreaks such as polio. This method is quick, sensitive and cost-effective and is being investigated as an option for monitoring the prevalence, trend and circulation of Coronavirus Disease 2019 (COVID-19) pandemics at the population level [17,18,19,20,21,22,23,24,25] and as an efficient early warning tool [17,26,27].

The aim of this study was to assess the feasibility and accuracy of a wastewater-based epidemiological monitoring tool in a hot spot/pandemic-affected area with a relatively high number of infection cases with SARS-CoV-2 in Transylvania/Romania/Europe, in the Sibiu City metropolitan area, namely to highlight the correlation between the number of infections on the days of sampling and the amount of virus RNA detected in wastewater. In order to accomplish this, we worked with the local municipal wastewater treatment plant, which provided the water samples. The Lucian Blaga University—Applied Ecology Research Center eco-toxicology and molecular biology laboratory performed the virus concentrations and viral RNA detection. The results showed that we could detect viral RNA in wastewater samples and the levels of viral nucleic acids mirror viral infections in the area. Moreover, the increase in viral nucleic acids occurs ahead of reported increased human infections, potentially due to a lag in reporting time. The results show that WBE is a reliable tool for assessing, monitoring and forecasting the status of the COVID-19 pandemic, especially in a climate of no restrictions and reduced patient testing.

## 2. Materials and Methods

### 2.1. Sample Collection and Virus Concentration

Samples were collected weekly, every Wednesday from 4 August 2021 to 2 March 2022, with three exceptions due to national holidays (1 December 2021, 29 December 2021 and 5 January 2022). Every week, the laboratory received a two-litre 24 h composite sample that was collected from the influx into the wastewater treatment plant that serves the municipality of Sibiu. In order to obtain the 24 h composite sample, workers at the wastewater treatment plant collect a 600 mL sample (proportional to the 645 L/s influx flow) every hour and then combine all 24 samples collected in one day. Two litres of that composite sample were transported to the laboratory, and on the same day, the virus was concentrated using the WHO “Guidelines for environmental surveillance of poliovirus circulation” protocol [28], which uses a standard procedure for the concentration of sewage specimens by the two-phase separation method. The following reagents were used: Dextran from *Leuconostoc* sp., molecular weight 40,000 (Sigma-Aldrich, St. Louis, MO, USA), Poly(ethylene glycol) (PEG) Mn 6000 (Sigma-Aldrich) and sodium chloride MW 58.44 g/mol (Sigma-Aldrich). For virus concentration, we used 500 mL of sample, as recommended in the protocol, mixed with 35 mL 5 N NaCl, 39.5 mL of 22% dextran and 287 mL of 29% PEG. The sample was incubated for 1 h at room temperature with constant agitation on a horizontal shaker. After mixing, the sample was transferred to a conical one-litre separation funnel set on a stand and allowed to precipitate/settle overnight at 4 °C. The lower layer was collected drop-wise in a sterile tube, amounting to a volume of about 5 mL per 500 mL of the original sample. The collected sample was extracted with 20% chloroform, and after centrifugation, the aqueous upper fraction was transferred to a cryotube; 280 μL of the sample was used for RNA extraction, and the rest was stored in a freezer at −80 °C. In order to confirm that we are able to recover the virus from water by this concentration method, we added 1 mL of nasopharyngeal sample from an infected patient to 500 mL of water and concentrated the virus, as described above.

### 2.2. RNA Extraction and Quantification

RNA was extracted from 280 μL of the sample using the QIAamp viral RNA extraction kit (Qiagen, Hilden, Germany) following the manufacturer’s recommendations. For each sample, two columns were used; 140 μL of the sample was extracted on each column as recommended by the manufacturer. The RNA from both columns was eluted in a final volume of 60 μL elution buffer. In vitro-made MS2 RNA was introduced in the sample lysis buffer and served as a control for the RNA extraction step and for the lack of inhibitors in the RT-qPCR reactions. The presence and quantity of the N (nucleocapsid), S (spike) and Orf1ab SARS-CoV-2 viral genes and the MS2 control were quantified by reverse-transcription quantitative PCR using the TaqPath COVID-19 CE-IVD RT-PCR kit (Thermo-Fisher Scientific, Waltham, MA, USA) following the manufacturer’s recommendations on the QuantStudio 5 Real-Time PCR System (Applied Biosystems, Waltham, MA, USA). The reverse transcription and quantitative PCR were performed in the same tube using the following reaction mix: 6.25 μL TaqPath™ 1-Step Multiplex Master Mix (No ROX™) (4X), 1.25 μL COVID-19 Real-Time PCR Assay Multiplex, 7.5 μL nuclease-free water and 10 μL RNA. Cycling conditions were: 2 min at 25 °C for UNG incubation, 10 min at 53 °C for reverse transcription, 2 min at 95 °C for activation, and 40 cycles of 3 s at 95 °C for denaturation and 30 s at 60 °C for annealing and extension, as recommended by the manufacturer (Thermo-Fisher Scientific). The primers and probes in the kit were designed and validated by Thermo-Fisher Scientific and approved to be used for the detection of nucleic acid from SARS-CoV-2. Samples were considered positive for N, S or Orf1 ab gene targets if the threshold quantification cycle (Cq) was lower than 40 cycles. The limit of detection for the three primer and probe sets found in the TaqPath COVID-19 CE-IVD RT-PCR kit was determined by the producer to be 10 copies of viral RNA per reaction. We obtained similar results in our lab. The TaqPath COVID-19 control (Thermo-Fisher Scientific), which has known concentrations of viral genes, was used to make standard curves for absolute quantification of the three viral genes in the samples (R^2^ for each curve was 0.99).

The daily number of infections in the Sibiu municipality was obtained from the local council’s official website (https://sb.prefectura.mai.gov.ro, accessed on 8 March 2022).

### 2.3. Statistical Analysis

The Shapiro–Wilk test was used to test the normality of the data in R. Because we obtained normal distribution of the data (*p* > 0.05) only when the viral copies per litre and the number of infections were log10 transformed, we used the log10 transformed values to make graphs and test for correlations. Graphs showing the number of reported infections and the amount of virus detected in WWTP influent were plotted in R using the ggplot2 package. Pearson correlation coefficients between the number of infections and viruses detected in sewage were calculated in R [29].

## 3. Results

**Figure 1:**
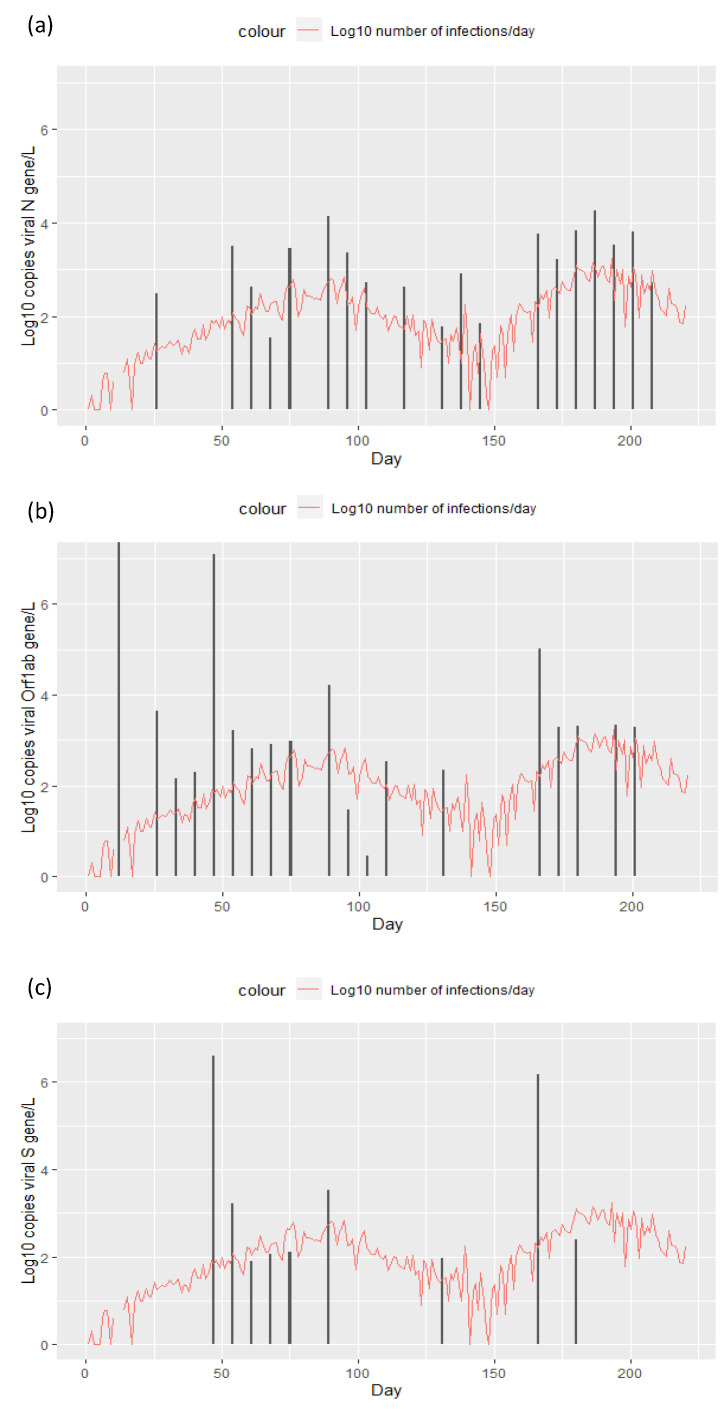
Log10 transformed number of daily reported SARS-CoV-2 infections in the Sibiu municipality between August 2021 and March 2022 (red line), and the log10 tranformed amount of viral RNA found in wastewater sampled once a week and quantified based on the presence of the viral genes (**a**) N gene, (**b**) Orf1 ab gene and (**c**) S gene (black bars).

The presence of SARS-CoV-2 viral RNA in water from the wastewater treatment plant serving the municipality of Sibiu was monitored once weekly over the course of 8 months starting in August 2021. The virus was concentrated by the two-step precipitation method recommended by WHO and quantified by RT-qPCR. The number of viral RNA copies for N, S and Orf1 ab genes was measured based on a standard curve. Figure 1 shows the log10 transformed amount of viral genes in parallel with the log10 transformed number of reported infections in the area. The figure shows that RNA for the N gene best reflects the trend of viral infections (Figure 1a), with Orf1 ab being the second best (Figure 1b), while the S gene was mainly detected before the start of a new wave of infections (Figure 1c).

**Figure 2:**
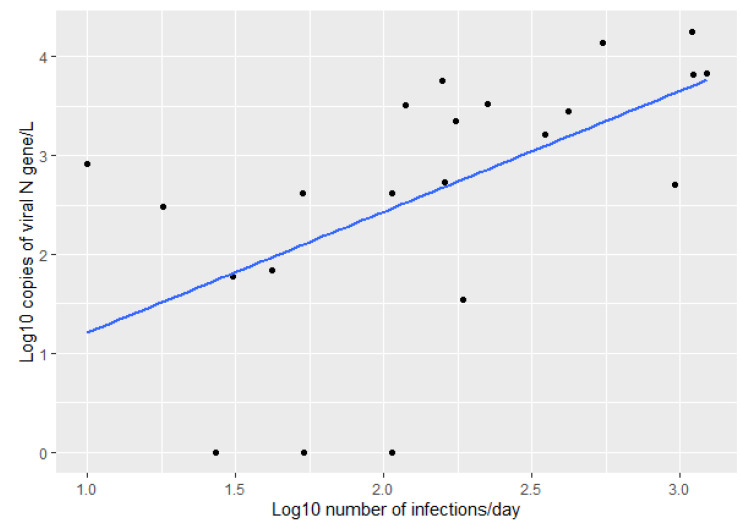
Correlation between the log10 transformed number of infections on the day of sampling and the log10 amount of virus detected in WWTP influent (Pearson correlation = 0.569).

As the virus in the wastewater comes from infected people, it is expected that an increased number of infections to lead to an increased concentration of virus in the wastewater. Therefore, we measured the correlation between the log10 transformed number of infections on the sampling day and the log10 transformed amount of virus, as measured by the quantity of N gene in the sample. Figure 2 shows a positive correlation between the amount of viral N gene in wastewater and the number of infections daily, with a Pearson correlation coefficient of 0.569.

**Figure 3:**
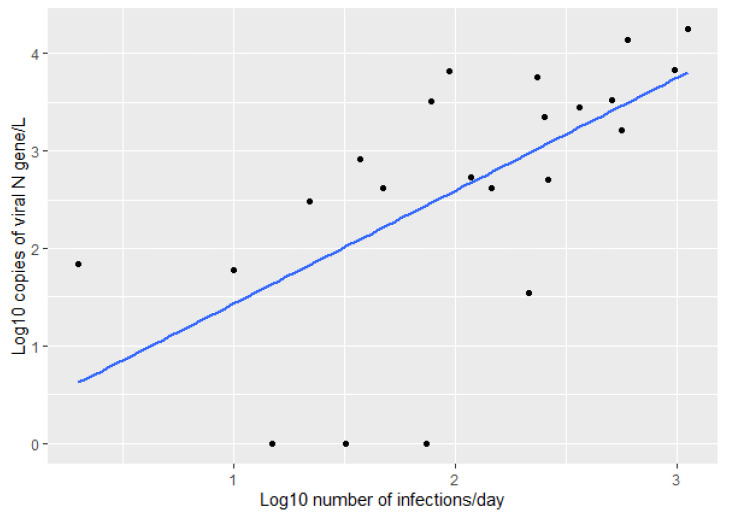
Correlation between the log10 transformed number of infections three days from the sampling day and the log10 transformed amount of virus detected in WWTP influent (Pearson correlation = 0.621).

Interestingly, when we performed the same correlation between the log10 transformed amount of virus in wastewater and the log10 transformed number of infections in the days following the sampling day, we found the Pearson correlation coefficient increased. We found the best correlation between the virus in the wastewater and the number of reported infections three days from the sampling day (Pearson coefficient = 0.621) (Figure 3).

**Figure 4:**
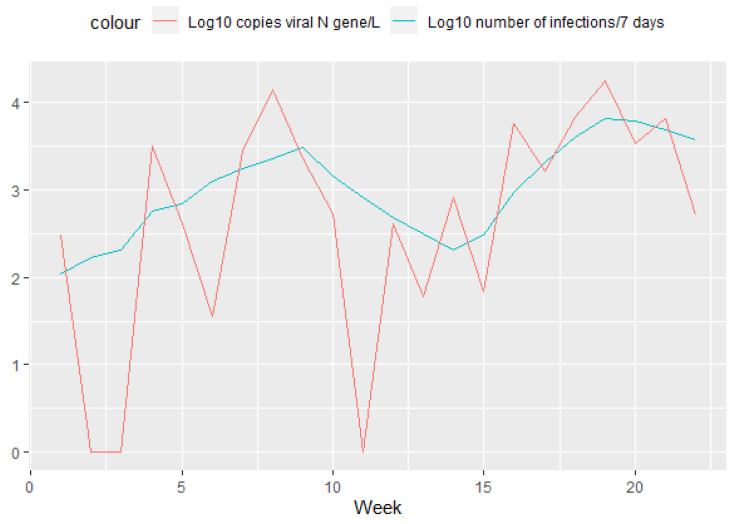
Log10 transformed amount of viral nucleic acid (N gene) detected in WWTP influent (red line) and the log10 transformed total number of infections that occurred during the week before sampling (blue line).

As the virus takes time to travel from individual households to the wastewater treatment plant, we were also interested in comparing the number of infections that occurred in the week up to the sampling day and the amount of virus found in wastewater. When infections are plotted as accumulated numbers over a 7-day period, we can clearly see the two infection waves that occurred in this study’s time interval (Figure 4 blue line). The first wave, triggered by the Delta variant of the virus, peaked at the beginning of November, while the second wave, caused by the Omicron variant, peaked at the beginning of February.

**Figure 5:**
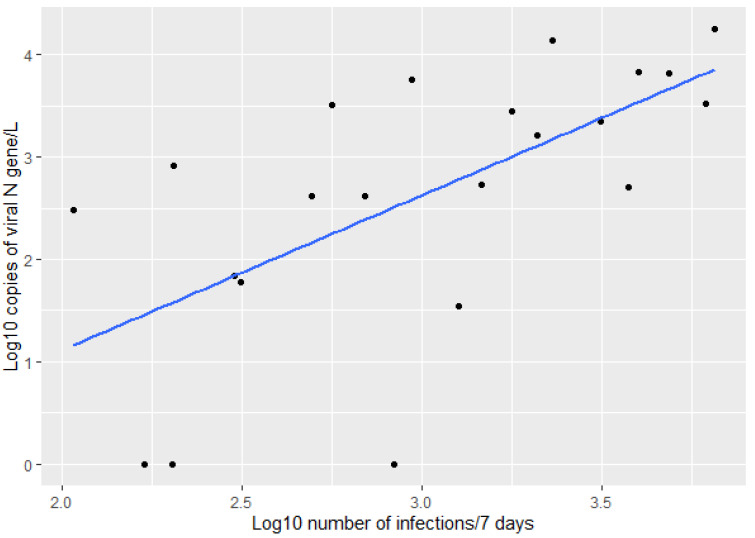
Correlation between the log10 transformed number of infections over the week previous to the sampling day and the log10 transformed amount of virus detected in WWTP influent (Pearson correlation = 0.632).

Once again, we can see a positive correlation between the number of infections and the amount of viral N gene found in wastewater (Figure 5, Pearson correlation coefficient 0.632).

**Figure 6:**
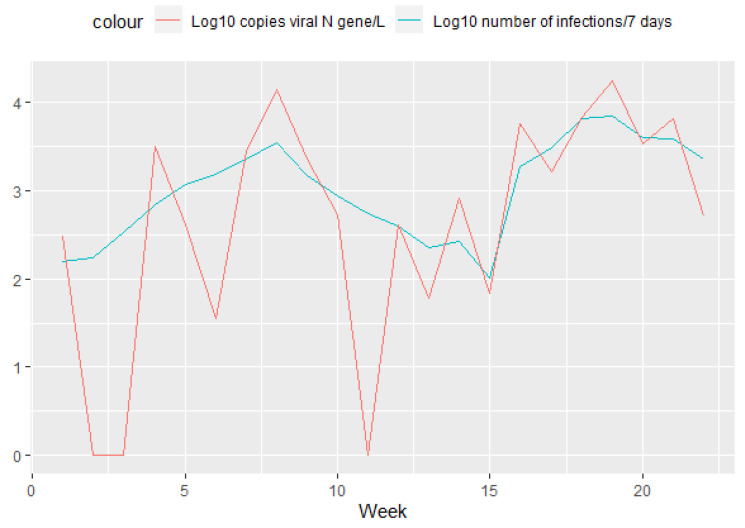
Log10 transformed amount of viral nucleic acid (N gene) detected in WWTP influent (red line) and the log10 transformed total number of infections that occurred during the week after the sampling (blue line).

**Figure 7:**
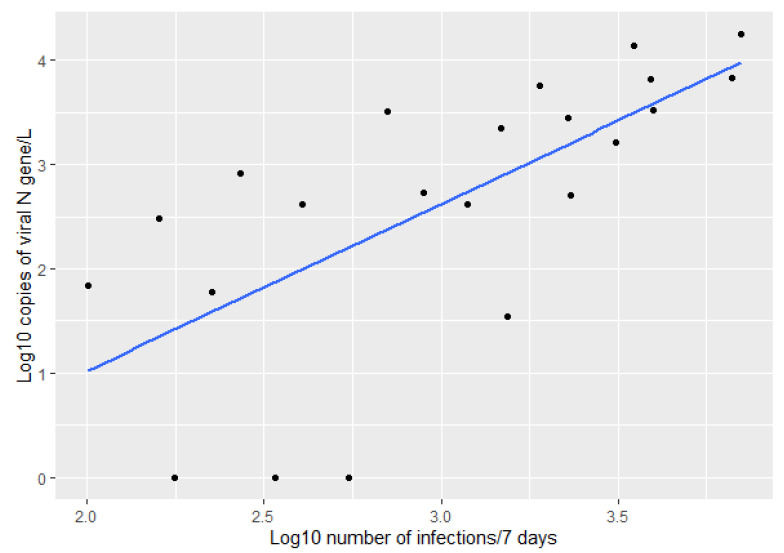
Correlation between the log10 transformed number of infections over the week starting on the sampling day and the log10 transformed amount of virus detected in WWTP influent (Pearson correlation = 0.681).

However, when we compared the amount of virus measured in WWTP influent with the number of infections reported in the 7 days following the sampling day, we found an even better correlation (Figure 6 and Figure 7, Pearson correlation = 0.681). This suggests that although the virus might take time to travel to the wastewater treatment plant, it takes even longer for authorities to report new infections. Therefore, this type of monitoring, based on wastewater, can catch an increase in infections ahead of the testing results.

## 4. Discussion

SARS-CoV-2 infection occurs in waves, as infection with one strain wains and a new viral strain starts infecting the population in a certain area. We monitored these waves in the Sibiu metropolitan area from August 2021 to March 2022. During this time period, the city experienced two infection waves, one with the Delta variant and the second with the Omicron variant. We monitored the status of SARS-CoV-2 infections by looking at the number of reported cases and by measuring the concentration of the virus in wastewater. The aim of the study was to see whether we could find a direct correlation between the concentration of the virus in wastewater and the number of reported cases.

In order to measure SARS-CoV-2 concentration in wastewater, we quantified the amount of three viral genes: N gene, S gene and Orf1 ab. In our opinion, the N gene was the best indicator of viral concentration in WWTP samples. Other studies also use the N gene as the preferred target for viral detection [30,31,32,33,34,35]. In our study, which aimed to quantify SARS-CoV-2 ARN in wastewater irrespective of the viral strain circulating at the time, the N gene is a logical best target as it is more conserved than the S gene.

We reported the SARS-CoV-2 viral amount in wastewater as concentration, viral gene copies per litre, and were able to show that the viral concentration is directly correlated with the reported number of infections. Other studies reported their data as concentration [34,36,37,38], and some even demonstrated that the normalization of clinical data using wastewater flow rate and percentage of sanitary flow did not significantly improve the correlation between the SARS-CoV-2 concentrations in wastewater and COVID-19 clinical cases in communities during the study period [33,39].

Sampling frequency varies greatly in similar studies, from daily sampling to studies where sampling was performed once a month [40,41,42]. However, it was suggested [43] that at least two samplings per week are required to obtain a good correlation between the number of infections and the amount of virus in the sewage sample. In this study, we showed a good correlation even at one sampling per week. Our weekly sampling very well captured the two infectious waves that occurred during the sampling period and mirrored the number of reported infections.

The peak of the virus in wastewater occurred before the peak of reported viral infections. A potential explanation for the more rapid detection of viral load increase in WBE compared with the increase in the number of infections is the delay in reporting the infections. Another cause could be the timing when people go to be tested. Usually, people are tested at the peak of infection when symptoms make it clear something is wrong with them. Viral shedding occurs before this; therefore, the virus in the wastewater increases before the number of reported cases increases. This delay in reporting the new cases makes WBE a more rapid way of gauging the rise of viral infections and, therefore, a helpful tool to monitor the pandemic.

Currently, there is no standardized protocol to detect and quantify SARS-CoV-2 in wastewater. A large interlaboratory study found that, depending on the viral concentration method and PCR platform used, the results can vary by several orders of magnitude [44], making it imperative that each laboratory validate its SARS-CoV-2 WBE method. In Romania, there is only one published study that looked at SARS-CoV-2 presence in wastewater [45]. However, that study only reported the ability to detect the presence of the SARS-CoV-2 virus in sewage samples. Therefore, our study is the first to test the feasibility of applying WBE in Romania. The results of this paper are timely, as all restrictions have been lifted as of 8 March 2022, leading to a marked decrease in testing and reporting of viral infections.

At the beginning of the SARS-CoV-2 pandemic, Romania declared a state of emergency and adhered to the strict rules and regulations meant to monitor and keep the pandemic in check. Among the most costly investments in the pandemic were to equip hospital laboratories to be able to test people for viral infection. Moreover, a nationwide informatic system was put in place to track the number of infections. However, as of 8 March 2022, the state of emergency ceased, and all restrictions were lifted. As a consequence, most of the mechanisms that were set in place at the beginning of the pandemic to monitor pandemic evolution and control it have now been cast aside, leaving authorities with little information about the real number of infections. The law no longer obligates people to test and declare their results. Moreover, there is no need to go to the hospital to be tested as there are easy-to-use antigen tests available in any pharmacy. Therefore, authorities and medical personnel no longer have a handle on the infection status of the population. However, the pandemic is not over, and the number of cases is surging in countries all over Europe, including Romania. In this situation, a method with low costs, rapid turnaround times, and reliability, such as wastewater-based epidemiology, should be considered a tool for public health surveillance and be included in the study area assessment and monitoring activities related to a new generation of threats and risks [46,47,48,49,50,51] for human and environmental health.

## 5. Conclusions

Wastewater-based epidemiology has long been used to track the status of pathogens such as polio. With the advent of the COVID-19 pandemic, it has been investigated as a way to monitor the SARS-CoV-2 spread. Studies show that WBE can be used as a reliable and cheap tool for monitoring virus spread trends in human communities and can help anticipate the rise and trend of new waves of viral infections.

This study aimed to test whether a WBE approach could be implemented in the municipality of Sibiu, Romania. The results show that the concentration of viral RNA in the wastewater is directly correlated with the number of infections reported in the week up to the sampling day and the number of infections reported for the sampling day. Moreover, the amount of viral RNA in wastewater increases in advance of the increase in officially reported infection cases, suggesting it could serve as a warning tool to alert authorities of a surge in infection numbers. From these results, we conclude that WBE can be used as a monitoring tool in our community, and this approach will allow the local and regional administration to impose timely adaptative measures for human population protection. Such a monitoring tool may be especially useful in the current climate where people are no longer compelled to test or quarantine.

## Data Availability

Data supporting reported results can be obtained at request from the co-authors of the paper.

## References

[1] Perlman S. (2020). Another decade, another coronavirus. N. Engl. J. Med..

[2] Fisher D., Wilder-Smith A. (2020). The global community needs to swiftly ramp up the response to contain COVID-19. Lancet.

[3] Zhu N., Zhang D., Wang W., Li X., Yang B., Song J., Zhao X., Huang B., Shi W., Lu R. (2020). A novel coronavirus from patients with pneumonia in China, 2019. N. Engl. J. Med..

[4] Lo I.L., Lio C.F., Cheong H.H., Lei C.I., Cheong T.H., Zhong X., Tian Y., Sin N.N. (2020). Evaluation of SARS-CoV-2 RNA shedding in clinical specimens and clinical characteristics of 10 patientswith COVID-19 in Macau. Int. J. Biol. Sci..

[5] Adhikari S.P., Meng S., Wu Y., Mao Y., Ye R., Wang Q., Sun C., Sylvia S., Rozelle S., Raat H. (2020). Epidemiology, causes, clinical manifestation and diagnosis, prevention and control of coronavirus disease (COVID-19) during the early outbreak period: A scoping review. Infect. Dis. Poverty.

[6] Lu J., du Plessis L., Liu Z., Hill V., Kang M., Lin H., Sun J., François S., Kraemer M.U.G., Faria N.R. (2020). Genomic epidemiology of SARS-CoV-2 in Guangdong Province, China. Cell.

[7] Peng L., Liu J., Xu W., Luo Q., Chen D., Lei Z., Huang Z., Li X., Deng K., Lin B. (2020). SARS-CoV-2 can be detected in urine, blood, anal swabs, and oropharyngeal swabs specimens. J. Med. Virol.

[8] Yan Y., Chang L., Wang L. (2020). Laboratory testing of SARS-CoV, MERS-CoV, and SARS-CoV-2 (2019-nCoV): Current status, challenges, and countermeasures. Rev. Med. Virol..

[9] Zheng S., Fan J., Yu F., Feng B., Lou B., Zou Q., Xie G., Lin S., Wang R., Yang X. (2020). Viral load dynamics and disease severity in patients infected with SARS-CoV-2 in Zhejiang province, China, January–March 2020: Retrospective cohort study. BMJ.

[10] Wölfel R., Corman V.M., Guggemos W., Seilmaier M., Zange S., Müller M.A., Niemeyer D., Jones T.C., Vollmar P., Rothe C. (2020). Virological assessment of hospitalized patients with COVID-2019. Nature.

[11] Lin L., Jiang X., Zhang Z., Huang S., Zhang Z., Fang Z., Gu Z., Gao L., Shi H., Mai L. (2020). Gastrointestinal symptoms of 95 cases with SARS-CoV-2 infection. Gut.

[12] Lehmann M., Allers K., Heldt C., Meinhardt J., Schmidt F., Rodriguez-Sillke Y., Kunkel D., Schumann M., Böttcher C., Sta-Hennig C. (2021). Human small intestinal infection by SARS-CoV-2 is characterized by a mucosal infiltratio with activated CD8+ T cells. Mucosal Immunol..

[13] Lee S., Yoon G.Y., Myoung J., Kim S.J., Ahn D.G. (2020). Robust and persistent SARS-CoV-2 infection in the human intestinal brush border expressing cells. Emerg. Microbes Infect..

[14] Arslan M., Xu B., El-Din M.G. (2020). Transmission of SARS-CoV-2 via fecal-oral and aerosols–borne routes: Environmental dynamics and implications for wastewater management in underprivileged societies. Sci. Total Environ..

[15] Gu J., Han B., Wang J. (2020). COVID-19: Gastrointestinal manifestations and potential fecal–oral transmission. Gastroenterology.

[16] Zhong M., Lin B., Pathak J.L., Gao H., Young A.J., Wang X., Liu C., Wu K., Liu M., Chen J.M. (2020). ACE2 and furin expressions in oral epithelial cells possibly facilitate COVID-19 infection via respiratory and fecal–oral routes. Front. Med..

[17] Medema G., Been F., Heijnen L., Petterson S. (2020). Implementation of environmental surveillance for SARS-CoV-2 virus to support public health decisions: Opportunities and challenges. Curr. Opin. Environ. Sci..

[18] Hart O.E., Halden R.U. (2020). Computational analysis of SARS-CoV-2/COVID-19 surveillance by wastewater-based epidemiology locally and globally: Feasibility, economy, opportunities and challenges. Sci. Total Environ..

[19] Ahmed W., Angel N., Edson J., Bibby K., Bivins A., O’Brien J.W., Choi P.M., Kitajima M., Simpson S.L., Li J. (2020). First confirmed detection of SARS-CoV-2 in untreated wastewater in Australia: A proof of concept for the wastewater surveillance of COVID-19 in the community. Sci. Total Environ..

[20] Sherchan S.P., Shahin S., Ward L.M., Tandukar S., Aw T.G., Schimitz B., Ahmed W., Kitajima M. (2020). First detection of SARS-CoV-2 RNA in wastewater in North America: A study in Louisiana, USA. Sci. Total Environ..

[21] Wang W., Xu Y., Gao R., Lu R., Han K., Wu G., Tan W. (2020). Detection of SARS-CoV-2 in different types of clinical specimens. JAMA.

[22] Gonzalez R., Curtis K., Bivins A., Bibby K., Weir M.H., Yetka K., Thompson H., Keeling D., Mitchell J., Gonzalez D. (2020). COVID-19 surveillance in southeastern Virginia using wastewater-based epidemiology. Water Res..

[23] Hillary L.S., Farkas K., Maher K.H., Lucaci A., Thorpe J., Distaso M.A., Gaze W.H., Paterson S., Burke T., Connor T.R. (2021). Monitoring SARS-CoV-2 in municipal wastewater to evaluate the success of lockdown measures for controlling COVID-19 in the UK. Water Res..

[24] Rusinol M., Zammit I., Itarte M., Fores E., Martinez-Puchol S., Girones R., Borrego C., Corominas L., Bofill-Mas S. (2021). Monitoring waves of the COVID-19 pandemic: Inferences from WWTPs of different sizes. Sci. Total Environ..

[25] Lundy L., Fatta-Kassinos D., Slobodnik J., Karaolia P., Cirka L., Kreuzinger N., Castiglioni S., Bijlsma L., Dulio V., Deviller G. (2021). Making waves: Collaboration in the time of SARS-CoV-2—Rapid development of an international co-operation and wastewater surveillance database to support public health decision-making. Water Res..

[26] Wu F., Xiao A., Zhang J., Moniz K., Endo N., Armas F., Bonneau R., Brown M.A., Bushman M., Chai P.R. (2020). SARS-CoV-2 titers in wastewater foreshadow dynamics and clinical presentation of new COVID-19 cases. mSystems.

[27] Ahmed W., Tscharke B., Bertsch P.M., Bibby K., Bivins A., Choi P., Clarke L., Dwyer J., Edson J., Nguyen T.M.H. (2021). SARS-CoV-2 RNA monitoring in wastewater as a potential early warning system for COVID-19 transmission in the community: A temporal case study. Sci. Total Environ..

[28] World Health Organization Guidelines for Environmental Surveillance of Poliovirus Circulation 2003. https://apps.who.int/iris/handle/10665/67854.

[29] R Development Core Team (2011). R: A Language and Environment for Statistical Computing.

[30] Shah S., Gwee S.X.W., Ng J.Q.X., Lau N., Koh J., Pang J. (2022). Wastewater surveillance to infer COVID-19 transmission: A systematic review. Sci. Total Environ..

[31] Pérez-Cataluña A., Chiner-Oms Á., Cuevas-Ferrando E., Díaz-Reolid A., Falcó I., Randazzo W., Girón-Guzmán I., Allende A., Bracho M.A., Comas I. (2022). Spatial and temporal distribution of SARS-CoV-2 diversity circulating in wastewater. Water Res..

[32] Acosta N., Bautista M.A., Waddell B.J., McCalder J., Beaudet A.B., Man L., Pradhan P., Sedaghat N., Papparis C., Bacanu A. (2022). Longitudinal SARS-CoV-2 RNA Wastewater Monitoring Across a Range of Scales Correlates with Total and Regional COVID-19 Burden in a Well-Defined Urban Population. Water Res..

[33] Zhao L., Zou Y., Li Y., Miyani B., Spooner M., Gentry Z., Jacobi S., David R.E., Withington S., McFarland S. (2022). Five-week warning of COVID-19 peaks prior to the Omicron surge in Detroit, Michigan using wastewater surveillance. Sci. Total Environ..

[34] Zheng X., Li S., Deng Y., Xu X., Ding J., Lau F.T., Yau C.I., Poon L.L., Tun H.M., Zhang T. (2022). Quantification of SARS-CoV-2 RNA in wastewater treatment plants mirrors the pandemic trend in Hong Kong. Sci. Total Environ..

[35] Layton B.A., Kaya D., Kelly C., Williamson K.J., Alegre D., Bachhuber S.M., Banwarth P.G., Bethel J.W., Carter K., Dalziel B.D. (2022). Evaluation of a Wastewater-Based Epidemiological Approach to Estimate the Prevalence of SARS-CoV-2 Infections and the Detection of Viral Variants in Disparate Oregon Communities at City and Neighborhood Scales. Environ. Health Perspect..

[36] Jakariya M., Ahmed F., Islam M.A., Al Marzan A., Hasan M.N., Hossain M., Ahmed T., Hossain A., Reza H.M., Hossen F. (2022). Wastewater-based epidemiological surveillance to monitor the prevalence of SARS-CoV-2 in developing countries with onsite sanitation facilities. Environ. Pollut..

[37] de Freitas Bueno R., Claro I.C.M., Augusto M.R., Duran A.F.A., Camillo L.D.M.B., Cabral A.D., Sodré F.F., Brandão C.C.S., Vizzotto C.S., Silveira R. (2022). Wastewater-based epidemiology: A Brazilian SARS-CoV-2 surveillance experience. J. Environ. Chem. Eng..

[38] Tanimoto Y., Ito E., Miyamoto S., Mori A., Nomoto R., Nakanishi N., Oka N., Morimoto T., Iwamoto T. (2022). SARS-CoV-2 RNA in Wastewater Was Highly Correlated with the Number of COVID-19 Cases during the Fourth and Fifth Pandemic Wave in Kobe City, Japan. Front. Microbiol..

[39] Ai Y., Davis A., Jones D., Lemeshow S., Tu H., He F., Ru P., Pan X., Bohrerova Z., Lee J. (2021). Wastewater SARS-CoV-2 monitoring as a community-level COVID-19 trend tracker and variants in Ohio, United States. Sci. Total Environ..

[40] Bertels X., Demeyer P., Van den Bogaert S., Boogaerts T., van Nuijs A.L., Delputte P., Lahousse L. (2022). Factors influencing SARS-CoV-2 RNA concentrations in wastewater up to the sampling stage: A systematic review. Sci. Total Environ..

[41] Huisman J.S., Scire J., Caduff L., Fernandez-Cassi X., Ganesanandamoorthy P., Kull A., Scheidegger A., Stachler E., Boehm A.B., Hughes B. (2022). Wastewater-based estimation of the effective reproductive number of SARS-CoV-2. Environ. Health Perspect.

[42] Feng S., Roguet A., McClary-Gutierrez J.S., Newton R.J., Kloczko N., Meiman J.G., McLellan S.L. (2021). Evaluation of sampling, analysis, and normalization methods for SARS-CoV-2 concentrations in wastewater to assess COVID-19 burdens in Wisconsin communities. ACS EST Water.

[43] Graham K.E., Loeb S.K., Wolfe M.K., Catoe D., Sinnott-Armstrong N., Kim S., Yamahara K.M., Sassoubre L.M., Mendoza Grijalva L.M., Roldan-Hernandez L. (2021). SARS-CoV-2 RNA in wastewater settled solids is associated with COVID-19 cases in a large urban sewershed. Environ. Sci. Technol..

[44] Pecson B.M., Darby E., Haas C.N., Amha Y.M., Bartolo M., Danielson R., Dearborn Y., Di Giovanni G., Ferguson C., Fevig S. (2021). Reproducibility and sensitivity of 36 methods to quantify the SARS-CoV-2 genetic signal in raw wastewater: Findings from an interlaboratory methods evaluation in the US. Environ. Sci. Water Res. Technol..

[45] Băicuș A., Cherciu C.M., Lazăr M. (2021). Identification of SARS-CoV-2 and Enteroviruses in Sewage Water—A Pilot Study. Viruses.

[46] Burcea A., Boeraş I., Mihuţ C.M., Bănăduc D., Matei C., Curtean-Bănăduc A. (2020). Adding the Mureş River Basin (Transylvania, Romania) to the list of Hotspots with High Contamination with Pharmaceuticals. Sustainability.

[47] Boeraş I., Burcea A., Coman C., Bănăduc D., Curtean-Bănăduc A. (2021). Bacterial microbiomes in the sediments of lotic systems ecologic drivers and role: A case study from the Mureş River, Transylvania, Romania. Water.

[48] Curtean-Bănăduc A., Burcea A., Mihuţ C.M., Bănăduc D. (2021). The benthic trophic corner stone compartment in POPs transfer from abiotic environment to higher trophic levels—Trichoptera and Ephemeroptera pre-alert indicator role. Water.

[49] Curtean-Bănăduc A., Burcea A., Mihuţ C.M., Berg V., Lyche J.L., Bănăduc D. (2020). Bioaccumulation of persistent organic pollutants in the gonads of Barbus barbus (Linnaeus, 1758). Ecotoxicol. Environ. Saf..

[50] Costea G., Pusch M.T., Bănăduc D., Cosmoiu D., Curtean-Bănăduc A. (2021). A review of hydropower plants in Romania: Distribution, current knowledge, and their effects on fish in headwater streams. Renev. Sust. Ener. Rev..

[51] Bănăduc D., Sas A., Cianfaglione K., Barinova S., Curtean-Bănăduc A. (2021). The role of aquatic refuge habitats for fish, and threats in the context of climate change and human impact, during seasonal hydrological drought in the Saxon Villages area (Transylvania, Romania). Atmosphere.

